# Changes in Molecular Characteristics of Cereal Carbohydrates after Processing and Digestion

**DOI:** 10.3390/ijms131216833

**Published:** 2012-12-10

**Authors:** Mirosław Marek Kasprzak, Helle Nygaard Lærke, Knud Erik Bach Knudsen

**Affiliations:** Department of Animal Science, Faculty of Science and Technology, Aarhus University, P.O. Box 50, 8830 Tjele, Denmark; E-Mails: hellen.laerke@agrsci.dk (H.N.L.); knuderik.bachknudsen@agrsci.dk (K.E.B.K.)

**Keywords:** dietary fiber, arabinoxylan, β-glucan, weight average molecular weight

## Abstract

Different extraction, purification and digestion methods were used to investigate the molecular properties of carbohydrates in arabinoxylan and β-glucan concentrates, dietary fiber (DF) rich breads and ileum content of bread fed pigs. The breads studied were: a low DF wheat bread (WF), whole meal rye bread (GR), rye bread with kernels (RK), wheat bread supplemented with wheat arabinoxylan concentrate (AX) and wheat bread supplemented with oat β-glucan concentrate (BG). The weight average molecular weight (M_w_) of extractable carbohydrates in β-glucan concentrate decreased eight-fold after inclusion in the BG bread when exposed to *in vitro* digestion, while the M_w_ of purified extractable carbohydrates in AX bread was reduced two-fold, and remained almost unaffected until reaching the terminal ileum of pigs. Similarly, the M_w_ of purified extractable carbohydrates in GR and RK bread was not significantly changed in the ileum. The AX bread resulted in the highest concentration of dissolved arabinoxylan in the ileum among all the breads that caused a substantial increased in ileal AX viscosity. Nevertheless, for none of the breads, the M_w_ of extractable carbohydrates was related neither to the bread extract nor ileal viscosity.

## 1. Introduction

Arabinoxylan and β-glucan naturally occur in the endosperm and aleurone cell walls of cereal grains with varying degrees of solubility. The content of rye and wheat arabinoxylan varies in the range of 6%–12% in the whole grain, and 21%–25% in the bran [1–3]. The proportion of β-glucan in whole grain oat is approximately 3% and 8% in the oat bran [4], whereas in the content of whole grain rye is reported to be ~1.0%–2.5% with double the amount in bran fractions [5]. Arabinoxylan is a random coil with varying degree of flexibility consisting of linked d-xylopyranosyl units with α-l-arabinofuranoside residues substituted at the 2- and 3-carbon position [6]. (1–3)(1–4)-β-d-glucan known as β-glucan is a linear glucosidic polysaccharides consisting of 60% 3-*O*-β-d-cellobiosyl-d-glucose, 30% 3-*O*-β-d-cellotriosyl-d-glucose, and 10% higher 3-*O*-β-d-cellooligosyl-d-glucose [7].

Due to the capability of soluble arabinoxylan and β-glucan to increase viscosity, they have physiologically gained a lot of interest as polysaccharides that can attenuate blood glucose and insulin responses and lower blood cholesterol [2,8,9]. Furthermore, the viscosity-elevating properties of arabinoxylan and β-glucan have been shown to slow the rate of gastric emptying and reduce the motility of human small intestine, which may reflect a prolongation of satiety [10]. Raised gastrointestinal viscosity can also reduce the rate of digestion and absorption of macronutrients, including fat, protein and carbohydrates [8,10] which may have a different effect on weight management [11,12].

Several physical, chemical and enzymatic methods have been reported in the literature to extract soluble arabinoxylan and β-glucan from foodstuffs such as grains, flour, rice, breads or pasta. The most common procedures are enzymatic, alkaline or water extraction with varying temperature and incubation time [4]. Boiling the sample for 15 min in 50% ethanol stabilizes the extract and inactivates endogenous or microbial enzymes [4]. Moreover, it allows the removal of lipid and enhances the efficiency of DF extraction. Water extraction at low temperature, however, brings a risk of insufficient extraction and solubilization of DF from the complex DF matrix, and increases the risk of aggregation in the water solution [8]. On the contrary, alkaline extraction enhances the DF solubilization and prevents aggregation [4] resulting in higher recovery than water- and enzymatic extraction procedures. Enzymatic and alkali treatments are used to break the ester and other covalent and/or non-covalent linkages between components and release the initially unextractable polysaccharides from the complex network of the cell walls grains [13]. Despite differences in the type of method of DF extraction, there are some general structural features that affect the extractability and solubility of DF: branching, glycosidic bonds, ionizing groups, and non-uniformity in repeating structure [13].

The molecular weight of DF components, their structure, solubility and concentration strongly influence the viscosity of aqueous solutions [14]. The molecular weight varies from 0.04 to 9 × 10^6^ g/mol of arabinoxylan in wheat and rye, with a polydispersity index (PDI) of 1.7–2 in wheat, indicating a broad distribution [1,3]. For comparison, the molecular weight distribution of β-glucan ranges from 10^4^ to 5 × 10^6^ g/mol in rye [1] and 0.04 to 2.5 × 10^6^ g/mol in oat [15], with a polydispersity index among cereals of 1.2–3.1 [15]. The polydispersity index reflects heterogenity of sizes of molecules in a polymer population [3]. However, mixtures of different polymers with uneven distribution will also result in a higher PDI.

**Figure 1:**
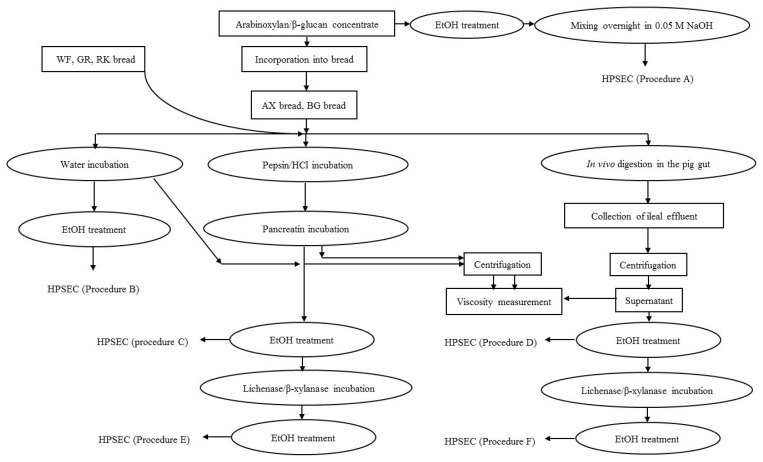
Overview of extraction, purification and sampling from fiber concentrates, bread extracts, and *in vitro* and *in vivo* digested bread for molecular weight determination.

During mixing, yeast fermentation and baking the DF polysaccharides provided as ingredients in the flour may change regarding molecular weight distribution and polydispersity [1]. Changes in the molecular weight and concentration of soluble DF influence the extract viscosity, which may have an impact on the functional properties of the breads and thereby on the physiological responses when consumed by monogastric mammals. As the gastrointestinal tract and physiological responses of pig are very similar to humans, the pig was used as a monogastric model providing access to digestive material that is difficult to obtain from humans. The aim of this investigation was to study the fate of polysaccharides in breads with variable content and structure of fiber constituents before and after *in vitro* and *in vivo* pig small intestinal digestion using different extraction procedures as illustrated in Figure 1.

## 2. Results

### 2.1. Content of Polysaccharides in DF Concentrates, DF Rich Breads and Ileal Supernatants

The soluble arabinoxylan concentrate used to produce AX bread had 31.2% total NSP and 23.4% arabinoxylan with an arabinose to xylose ratio (A/X) of 0.94. The soluble oat β-glucan incorporated into the BG bread had 40.5% total NSP and 35.2% β-glucan. The arabinoxylan and β-glucan concentrate contained 4.0% and 41.8% of starch, respectively.

The starch content in the fiber rich breads varied from 50.9% to 60.8% (Table 1) compared to 71.1% in the WF bread. The NSP content of the breads varied from 11.2% to 16.7% in the fiber rich breads, whereas the low fiber bread (WF) had only 3.5% NSP. A large proportion of insoluble NSP in the BG bread consisted of cellulose, which was added in order to equilibrate the DF content among the high DF breads. Although the content of arabinoxylan practically was the same in the GR, RK and AX breads, 87% of the arabinoxylan in the AX bread was soluble compared to only 47%–48% in the GR and RK breads. The solubility of β-glucan in the BG, GR, and RK bread were 82%, 29%, 21%, respectively.

The concentration of starch was very low (<0.9 mg/mL) in the ileal supernatant from pigs fed the arabinoxylan rich breads, while the content in ileal supernatant from the BG fed pigs was substantially higher with 2.7 mg/mL (Table 1). The concentration of NSP in ileal supernatant ranged from 10 to 20 mg/mL in pigs fed WF, GR, RK or BG, whereas the concentration in the ileal supernatant of AX fed pigs was 56.3 mg/mL. Most of the NSP in ileal supernatants of the WF, GR, RK and AX fed pigs comprised of arabinoxylan (>78%). Surprisingly 50% of the NSP in the ileal supernatant of BG fed pigs also consisted of arabinoxylan and only 26% of NCP-glucose. The A/X was the same in the ileal effluent of the AX fed pigs as in the bread, while the A/X increased slightly in the ileal supernatant of pigs fed WF, GR and RK bread. In contrast, the A/X markedly decreased from 1.37 in the BG bread to 1.04 in the ileal supernatant.

### 2.2. Molecular Properties of the Wheat Arabinoxylan and Oat β-Glucan Concentrates

**Figure 2:**
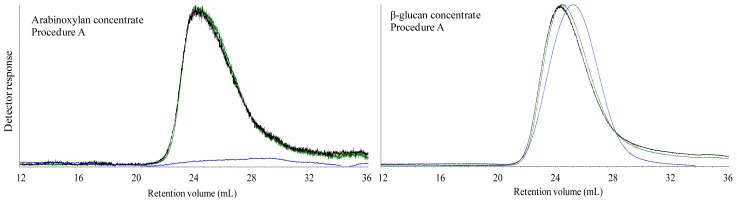
Size exclusion chromatography with RALLS (green), LALLS (black) and intrinsic viscosity (blue) signal of alkaline extracts of fiber concentrates (procedure A).

The M_w_ of the alkaline extract of the fiber concentrates (Procedure A) were 602 kDa for the arabinoxylan and 1978 kDa for the β-glucan concentrate. The signals of right angle laser light scattering (RALLS), low angle laser light scattering (LALLS) and intrinsic viscosity (IV) of β-glucan concentrate closely overlapped with a Gaussian type distribution (Figure 2). The alkaline extract of the arabinoxylan concentrate showed a low and flatted IV signal, whereas RALLS and LALLS signals followed each other.

### 2.3. Molecular Characteristics of Water Extracted and *in vitro* Digested Breads

The M_w_ of extractable carbohydrates in water extracts (procedure B) of all breads were significantly higher than after *in vitro* digestion (procedure C). After water extraction the majority (>83%) of carbohydrates had an M_w_ above 200 kDa in the WF, GR, and RK breads, while approximately half the carbohydrates was above 200 kDa in the two breads containing AX and BG concentrates (Table 2). After *in vitro* digestion, in addition to a dramatic M_w_ decrease in all breads, the fraction below 200 kDa also became dominant in the control bread and all arabinoxylan rich breads (WF, GR, RK, and AX), while the BG bread still had a similar proportion of molecules above and below 200 kDa, respectively. Simultaneously, in all cases except for BG, *in vitro* digestion also led to a higher polydispersity indicating a broader distribution of extracted molecules.

**Figure 3:**
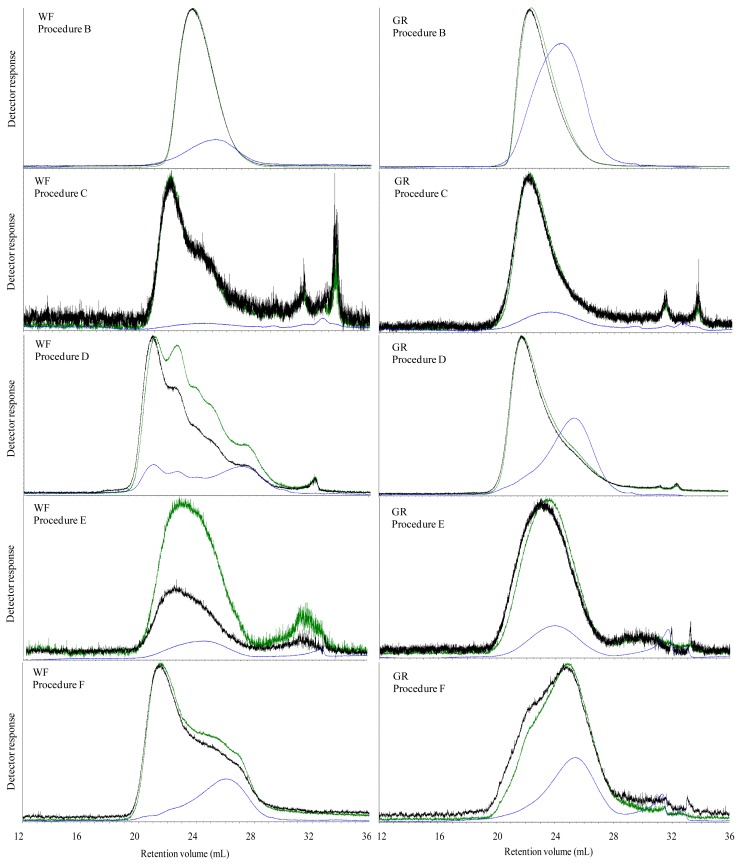
Size exclusion chromatography with RALLS (green), LALLS (black) and intrinsic viscosity (blue) signal of extracts of water extracted (procedure B), *in vitro* digested (procedure C), *in vivo* digested (procedure D) and lichenase treated *in vitro* digested (procedure E) and lichenase treated *in vivo* digested (procedure F) WF and GR breads.

**Figure 4:**
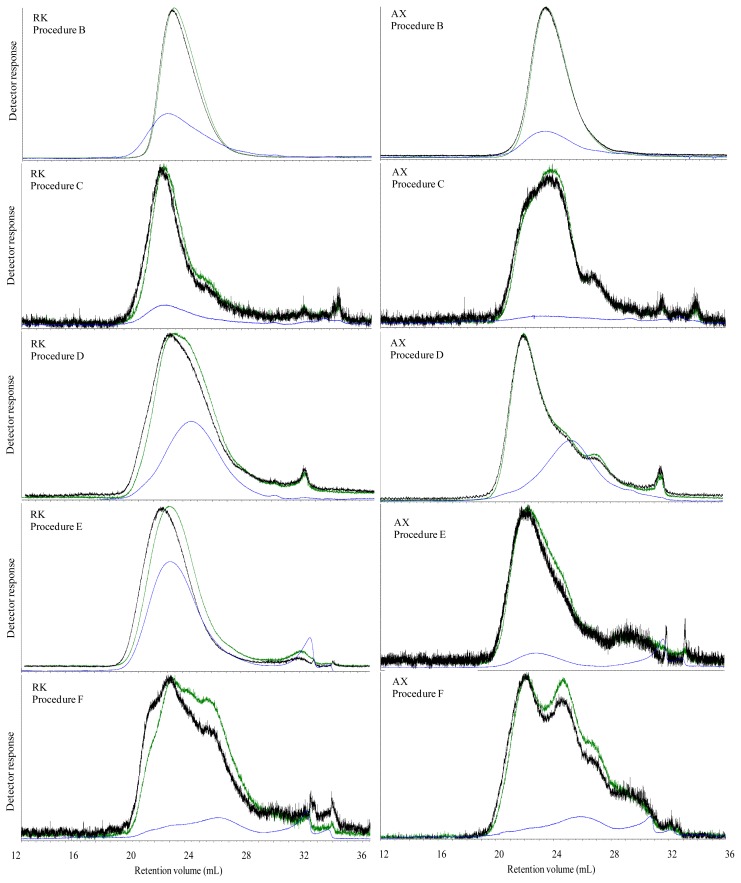
Size exclusion chromatography with RALLS (green), LALLS (black) and intrinsic viscosity (blue) signal of extracts of water extracted (procedure B), *in vitro* digested (procedure C), *in vivo* digested (procedure D) and lichenase treated *in vitro* digested (procedure E) and lichenase treated *in vivo* digested (procedure F) RK and AX breads.

**Figure 5:**
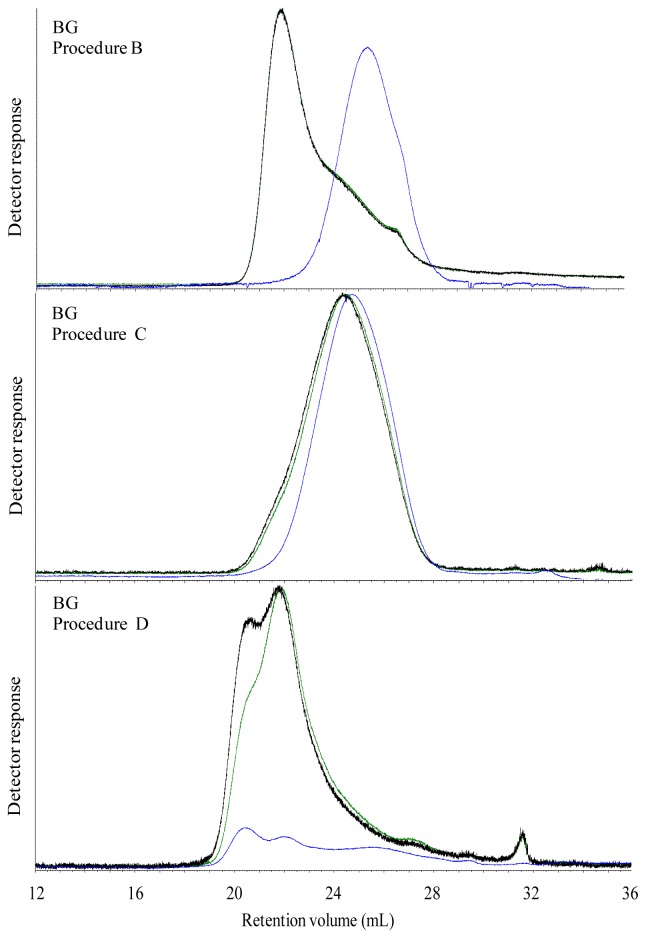
Size exclusion chromatography with RALLS (green), LALLS (black) and intrinsic viscosity (blue) signal of extracts of water extracted (procedure B), *in vitro* digested (procedure C), and *in vivo* digested (procedure D) BG bread.

The elution profile of water extractable carbohydrates in WF and GR breads (Figure 3), RK and AX bread (Figure 4) displayed a unimodal distribution of molecular weight, while the elution profile of water extracted BG (Figure 5) was polymodal with two-three different populations. The elution profile of water extractable carbohydrates from the GR and BG bread show a high ratio of RALLS and LALLS to IV signal of the early-eluting polymers, and a high IV response later in the chromatogram. *In vitro* enzymatic extracts of WF, GR, RK and AX breads showed a flatted IV signal. The RALLS and LALLS elution profile did not change markedly in GR and RK breads after *in vitro* digestion compared with their water incubated counterparts, although a long tail was observed in RK (Table 2). The RALLS and LALLS elution profile of *in vitro* incubated AX bread showed two populations eluting at 18–25 mL and a small population eluting at 26 mL (Figure 4). The RALLS and LALLS elution profile of *in vitro* incubated WF bread showed two populations in the 18–28 mL range. The elution profile of RALLS, LALLS and IV signal of *in vitro* incubated BG bread overlapped, indicating unimodal distribution (Figure 5).

### 2.4. Molecular Characteristics of Ethanol Precipitated Digesta Supernatants

The molecular properties of extracted carbohydrates from ileal materials without further purification (Procedure D) is shown in Table 3. M_w_ of ethanol treated ileal supernatant of pigs fed GR, RK, AX and BG was 335–397 kDa, whereas ethanol treated supernatant of WF had a M_w_ of 259 kDa. The proportion of the polymer population above 200 kDa was below 21.2% among all ileal supernatants. Compared to *in vitro* digestion a higher IV signal was observed by *in vivo* digestion (Figures 3 and 4). The elution profile showed polymodal molecular weight distribution in WF and AX ileal supernatants, indicating approximately five and three population, respectively. The ileal supernatants of GR and RK displayed an unimodal distribution, whereas ileal supernatant of BG fed pigs was at least bimodal.

### 2.5. Molecular Characteristics of Lichenase Treated *in vitro* and *in vivo* Digested Breads

Lichenase was used to remove β-glucan from the re-dissolved polysaccharides in order to get a more pure arabinoxylan peak. β-xylanase was also used to purify β-glucan from the digested BG breads. However, unfortunately, the β-xylanase used turned out to have β-glucanase side activity. Therefore, only results of *in vitro* and *in vivo* digested BG bread without further purification are presented here.

The lichenase purified extractable carbohydrates in the *in vitro* digestion (Procedure E) resulted in the lowest M_w_ of 219 and 250 kDa in WF and GR bread extracts and the highest M_w_ of 514 kDa in RK bread extract (Table 4). Similarly, the purified extractable carbohydrates in the *in vivo* digested bread (Procedure F) showed the lowest M_w_ of 265 kDa in ileal supernatant of GR fed pigs and the highest M_w_ of 419 kDa in ileal supernatant of pigs fed RK (Table 5). However, for M_w_ was 360 kDa in the *in vivo* digested WF when samples were purified with lichenase. The M_w_ of carbohydrates in the lichenase purified *in vitro* digested and *in vivo* digested AX bread was very similar with M_w_ of 307 and 315 kDa, respectively.

The elution profile of the lichenase purified *in vitro* digested breads (procedure E) showed an unimodal distribution of molecules in GR and RK, whereas AX and WF displayed broadening and shoulder appearance indicating polymodal distribution of extracted polymers. In contrast, the elution profile of lichenase purified *in vivo* digested RK and AX ileal supernatants (procedure F) showed at least three populations, whereas the elution profile of lichenase purified ileal supernatants of WF and GR fed pigs indicated at least two populations.

### 2.6. Viscosity of Water Extracted, *in vitro* and *in vivo* Digested Breads

Water incubation as well as *in vitro* and *in vivo* digestion of breads led to differences in extract viscosity (Table 6). Generally, except for BG, water extracts resulted in lower extract viscosity than *in vitro* digested breads, which again were lower than the viscosity of the ileal supernatant from the bread fed pigs.

Of the water extracts BG bread showed the highest viscosity (10.7 mPa.s), while the lowest viscosity was in water extracts of AX and WF bread (1.8 and 1.3 mPa.s). Under simulated *in vitro* digestion, highest extract viscosities were also obtained with BG, followed by RK and GR and the lowest in AX and WF. Viscosity of the ileal supernatant of AX fed pigs was markedly higher than with the other breads, whereas the viscosity of the ileal supernatant of the BG fed pigs was significantly lower.

## 3. Discussion

The potential of a polymer to increase the viscosity, in our case in the gastrointestinal tract, is affected by the molecular weight, concentration, structure of polymer, and possible interaction with other molecules. Hence, not only the properties of the fiber source itself, but also changes that occur during processing and in the digestive tract may affect the physicochemical properties of the fiber. To elucidate this, we have used different procedures to extract and purify extracts of bread and studied fiber modification after baking and passage of the small intestine.

The M_w_ of alkaline extract of fiber concentrates (602 kDa for arabinoxylan, 1978 kDa for β-glucan) were in line with other investigations. Saulnier *et al.*[3] referred to M_w_ of wheat arabinoxylan varying from 70 kDa to 655 kDa, while Åman *et al.*[14] characterized oat β-glucan indicating Calcofluor average molecular weight 2060–2300 kDa. The studied fiber concentrates were extracted under mild alkaline conditions and showed a high M_w_ that was caused by the extractable fiber, but possibly also some starch residues. The main reason of using an alkaline procedure was to prevent aggregation of β-glucan and arabinoxylan [16] as well as β-elimination or peeling reactions (sequential elimination of the reducing end residues from the polysaccharides chain) than may occur at a high pH [17]. Although the manufacture of both DF concentrates included a starch elimination step using α-amylase and amyloglucosidase [18], particularly the β-glucan concentrate still contained high amounts of starch residues that might have interfered in the M_w_ determination. On the other hand, the unimodal shape of the BG chromatogram suggests that this was not the case, probably because remaining starch was heavily degraded by the enzymatic treatment during manufacturing.

The M_w_ of carbohydrates was high in all water extracted breads, which most likely was caused by dissolution of both starch and DF. However, in spite of an apparent high M_w_ of water extractable carbohydrates, the viscosity of the water extract were low in all breads with exception of BG bread. This was possibly due to an insufficient extraction of DF from the bread matrix, indicating that polymers of gelatinized starch and DF present in the grain matrix in the bread were linked with each other or other bread components. The high viscosity of water extract of BG bread was due to the linear structure of β-glucan, which most likely favor its extraction and solubilization in water solution and thereby increasing the viscosity.

The aim of treating the breads with digestive enzymes was to simulate the digestive processes in the gut, but simultaneously this digestion step was expected to deplete DF fraction of the bread for starch and protein. Addition of digestive enzymes did not affect the viscosity of BG bread, whereas the viscosity of WF, GR, RK and AX bread increased after *in vitro* digestion compared to water extracts. This might suggest that complex structure of arabinoxylan either present in grain matrix or added as concentrate require hydrolysis of starch and protein to enhance its extractability. Moreover, *in vitro* digestion overall caused a marked decrease in M_w_ compared to the water extracts in the all of breads. Since none of the enzymes used for *in vitro* digestion are able to degrade DF polymers, the low M_w_ of *in vitro* digested bread compared to the water extracts was possibly due to removal of starch and protein from the matrix. Moreover, the very low M_w_ of extractable carbohydrates from the AX bread after *in vitro* digestion (134 kDa) might be due to a low extraction efficiency of branched wheat arabinoxylan polymers (A/X 0.97) in water, resulting also in a low viscosity (2.5 mPa.s) compared to the GR and RK breads. Lichenase purification had a minor impact on the estimated average M_w_ although it increased in the *in vitro* digested RK and AX breads. This was consistent with a removal of low M_w_ components as the proportion being lower than 200 kD decreased for all breads after lichenase treatment but particularly for these two breads.

By inclusion of wheat arabinoxylan concentrate in wheat bread, we observed that the M_w_ of purified extractable carbohydrates in AX bread was reduced two-fold (307 kDa) compared to the arabinoxylan concentrate (602 kDa). Andersson *et al.*[1] reported that arabinoxylan whole rye flour had a M_w_ of 4 × 10^4^–9 × 10^6^ Da with an average of 2 × 10^6^ Da, and arabinoxylan whole grain breads showed more narrow M_w_ of 6 × 10^5^–9 × 10^5^ Da. This is in the same range as the lichenase purified *in vitro* digested RK bread (514 kDa), but much higher than the M_w_ of in the WF, GR and AX breads. The reduction in M_w_ of rye and wheat arabinoxylan during bread making is mainly caused by microbial endoxylanases and to minor extent by endogenous endoxylanases present in wheat and rye kernels [17,19,20]. Besides enzymatic degradation, arabinoxylan is sensitive to physical and thermal processing such as mixing and heating, which may lead to degradation of arabinoxylan at 40–70 °C [17,21]. Similar to arabinoxylan, β-glucan also depolymerized during bread making. Depolymerization occurs during the mixing and proofing of bread dough with help of added yeast and β-glucanases present in rye flour [22]. Therefore, along with removal of starch, the M_w_ of extractable carbohydrates in *in vitro* digested BG bread was reduced eight-fold compared to the M_w_ in the β-glucan concentrate, as previously also demonstrated with barley β-glucan incorporated into wheat bread [23].

The M_w_ of extractable carbohydrates was higher in ileal supernatant of GR, RK, AX and BG fed pigs than of the *in vitro* digested breads (Tables 2 and 3). Again, this supports the view that *in vitro* digestion did not completely remove starch and protein digestion products from the extract. *In vivo*, most starch was digested [24] leaving a very low concentration in the ileal supernatant except for the BG fed pigs.

In our study, there was a marked change in the elution profile of components after *in vivo* digestion compared to *in vitro* digestion particularly in the lichenase purified samples. This resulted in the formation of three populations in the RK and AX extracts and two populations in WF and GR extracts. In spite of the change in elution profile, the M_w_ of the lichenase purified *in vitro* and *in vivo* digested breads remained almost unchanged (Tables 4 and 5). The upper gastrointestinal tract is inhabited by protozoa, fungi and cellulotytic and xylanolytic bacteria species, which are able to degrade a mixture of homo- and heteropolymer substrates, including xyloglucan, β-glucan, glucuronomanan, arabinoxylan and xylan [25,26]. A range of studies have also shown that both quantitatively and quantitatively β-glucan and arabinoxylan may be degraded in the distal small intestine of pigs and humans [27–31], but most studies points toward arabinoxylan being less susceptible towards degradation than β-glucan. This is due to the more complex structure of arabinoxylan, which presumably prevents disintegration by microbial enzymes, since seven different enzymes are required to cleave the arabinoxylan molecule [32]. Hence, the higher concentration of arabinoxylan in ileal supernatant of the AX fed pigs compared to the GR and RK fed pigs could be brought about by two counteracting mechanisms; higher solubility of the wheat arabinoxylan concentrate added to the wheat bread than of the arabinoxylan located in the cell walls of rye in the GR and RK bread, or lower degree of degradation of the added wheat arabinoxylan compared to the two rye breads. Although we did not find significant differences in the ileal digestibility of arabinoxylan between the breads, the ileal digestibility of the AX bread was numerically lower [24]. Furthermore, as the arabinoxylan in the AX bread was highly branched (A/X 0.97) compared to the arabinoxylan found in the two rye breads (A/X 0.70–0.74), we may expect that this branching pattern (*i.e.*, high xylose substitution by arabinose) protect the xylose chain toward extensive degradation by microbial enzymes and thereby allow a high concentration of arabinoxylan in the ileal supernatant, which also resulted in a significantly higher viscosity. In contrast to arabinoxylan, the linear structure of β-glucan was easily degraded by the microbial enzymes that led to a reduction in the ileal viscosity of BG bread. Nevertheless, the reduced M_w_ of extractable carbohydrate in ileal supernatants was not related to the ileal viscosity among all breads.

## 4. Experimental Section

### 4.1. Breads and Diets

The investigation included a control bread baked on white wheat flour (WF) and four iso-DF breads: two commercial whole grain rye breads without (GR) and with grain kernels (RK) and two experimental wheat flour based breads supplemented with arabinoxylan concentrate (AX) and β-glucan concentrate (BG), respectively. WF, GR and RK were commercial breads produced by Schulstad Lantmännen A/S (Hammerholmen, Hvidovre, Denmark) with the commercial names: Hvede Toast, Mørkt Rugbrød and Multikernerugbrød, respectively. The ingredients for WF were in decreasing order; wheat flour (68%), water, yeast, sugar, salt, vinegar, canola oil, emulsifier (E471, E472e), rye flour, barley malt flour, flour treatment agent (ascorbic acid); GR was baked on; rye whole meal, water, rye sourdough, rye bread crumps, salt, vinegar, dried sourdough rye, canola oil, yeast, barley flour, and RK was produced from: rye kernels (49%), water, rye sourdough, rye bread crumbs, wheat meal, salt, yeast, vinegar, dried sourdough rye, canola oil, rye four, barley flour. BG and AX breads were experimental breads baked at a local bakery (Konditor-Bageren, Ørum, Denmark). The ingredients for AX were; white wheat flour, arabinoxylan concentrate (wheat arabinoxylan, Manildra Group Ltd, Nowra, Australia), sugar, salt, margarine, and yeast. The BG bread was prepared from white wheat flour, β-glucan concentrate (oat β-glucan, known commercially as PromOat, BioVelop AB, Kimstad, Sweden), Vitacel WF 600 (16.9% of arabinoxylan, J. Rettenmaier & Söhne GmbH, Rosenberg, Germany), wheat gluten, sugar, salt, margarine, and yeast. The breads were cut into pieces, frozen at −20 °C and following mincing partly frozen. The minced breads were mixed with digestibility markers (chromic oxide and Celite^®^), vitamins, minerals and in case of AX bread with whey protein concentrate (Lacprodan 87; Arla Foods Ingredients Amba, Viby J, Denmark), in order to adjust the protein content of the diets. The feed was portioned into plastic bags containing 575 g DM per meal and stored at −20 °C until consumption.

### 4.2. Animal Study

The pigs used in the study were crossbreeds of Duroc × Danish Landrace × Yorkshire obtained from the swineherd at Aarhus University, Department of Animal Science, Denmark. The animal experiment was conducted according to protocols approved by the Danish Animal Experiments Inspectorate and complied with the guidelines of the Danish Ministry and Justice concerning animal experimentation and care of animals under study.

Five female pigs with average weight of 58 ± 2.8 kg were fitted surgically with a permanent simple T-cannula 15 cm anterior to the ileal-caecal junction following the procedure of Jørgensen *et al.*[33] and allowed to recover until start of the study. The pigs were fed each experimental diet for 1 week in a Latin Square design. Prior to the feeding, the diets were thawed. Total daily feed intake was 1725 g DM/d and drinking water was provided *ad libitum*.

On day 6 and 7 ileal effluent was collected continuously for 4 h after the morning meal. Ileal effluent was collected in 6 mm × 20 mm polyamide autoclave bags (Buch and Holm, Herlev, Denmark) attached to the ileum cannula with a plastic zip fastener. Two to three drops of an aqueous solution of 0.2% NaN_3_ (Sigma-Aldrich, St. Louis, MO, USA) was added to the collection bags to prevent microbial activity. Forty grams of material from each day of collection was centrifuged (10,000× *g*, 4 °C, 20 min) in 50 mL tubes. Following the supernatant from the 2 collection days was pooled into 10 mL tubes and stored at −20 °C until further analysis.

Results on carbohydrate digestibility and physicochemical properties of ileal effluent are described elsewhere [24].

### 4.3. Sample Preparation for HPSEC Analysis

The fiber concentrates, breads and the supernatants of ileal digesta were analyzed as illustrated in Figure 1 and described in detail below.

#### 4.3.1. Solubilization of DF from the Fiber Concentrates

Extraction of DF from the fiber concentrates was done according to the procedure of Suortti [4] illustrated in Figure 1 as Procedure A. One hundred milligrams of arabinoxylan and β-glucan concentrate were weighed into 50 mL plastic tubes. Five mL of 50% ethanol was added, then the sample was mixed, placed in a boiling water bath for 15 min and cooled. Additionally, 5 mL of 50% ethanol was added, mixed, centrifuged (1000× *g*) and the precipitate collected. Twenty milliliters of 0.05 M aqueous NaOH solution was added to the precipitate and mixed overnight at room temperature and filtered through a 0.2 μm minisart filter (Sartorius Stedim Biotech Gmbh, Germany) prior to HPSEC analysis.

#### 4.3.2. Water Extraction and *in vitro* Digestion of Breads

The breads were extracted in azid-containing water or subjected to *in vitro* enzymatic digestion according to Procedures B and C, as illustrated in Figure 1.

Prior to extraction, the breads were minced for 1 min using a hand blender (Braun, Kronberg, Germany) and then the material was weighed in triplicate into a tube adjusted to a DM content of 13% in the solution, in order to mirror the approximate final DM content of ileal digesta. For *in vitro* digestion (Procedure C), the breads were initially incubated with a 11.68 mL pepsin solution (12 mg, 3.260 U/mL, EC.3.4.2.3.1., Merck, Darmstadt, Germany) in 0.1 M HCl in a shaking water bath (150 strokes/min) at 39 °C for 2 h. Following pH was adjusted to 7.0 by addition of a 6.54 mL solution of porcine pancreatin (50 mg, 8 × USP, EC. 232-468-9, Sigma-Aldrich, St.Louis, MO, USA) in 1 M NaHCO_3_, and incubated in the same water bath for further 2 h. Water extraction (Procedure B) followed the same procedure except that the enzyme solutions were replaced with an aqueous solution of 0.02% NaN_3_ (Sigma-Aldrich, St. Louis, MO, USA). After the water and *in vitro* incubations, the bread mixtures were centrifuged and 5 mL of supernatants were transferred to a 50 mL plastic tube and treated with ethanol: Five milliliter of 96% ethanol was added to the supernatant, mixed, and placed in a boiling water bath for 15 min, cooled and supernatant was discarded. Another 5 mL of 50% ethanol was added, the sample was mixed, centrifuged (1000× *g*) and the precipitate collected. Five milliliters of 0.02% NaN_3_ was added to the precipitate and the mixture was boiled for 10 min and cooled. The samples were filtered and injected into the HPSEC system.

For a parallel set of samples the precipitate after *in vitro* digestion and subsequent ethanol treatment was further treated with either lichenase or β-xylanase in order to purify the arabinoxylan and the β-glucan, respectively (Procedure E). The precipitate of the BG bread was incubated with 50 μL of β-xylanase (from Trichoderma viride, 2300 U/mL, Megazyme International Ireland, Wicklow, Ireland) in 5 mL of 0.1 M sodium acetate buffer (pH 5) at 50 °C for 2 h, whereas precipitates of WF, GR, RK and AX breads were incubated with 100 μL of lichenase (from Bacillus subtilis, 1000 U/mL, Megazyme International Ireland) in 5 mL of 0.1 M sodium acetate and 2 mL of 0.5 M Na_2_HPO_4_ (added to increase pH to 6.5–6.7) at 60 °C for 2 h in the water bath. During the incubation, the samples were occasionally stirred. Afterwards, samples from both incubations were cooled and mixed with 40 mL of 96% ethanol in an ice box for 30 min. The samples were centrifuged (1000× *g*), and supernatants were discarded, while precipitates were re-dissolved in 5 mL of 0.02% aqueous NaN_3_ solution in a boiling water bath for 10 min. After cooling, the samples were filtered and analyzed by HPSEC.

The step of ethanol treatment in our procedures was previously described by Andersson *et al.*[1].

#### 4.3.3. Isolation and Purification of DF from Ileal Effluent

The ileal supernatant was thawed and 5 mL was transferred to a 50 mL plastic tube. Five milliliters of 96% ethanol and 5 mL of 50% ethanol were added to the supernatant and treated according to the ethanol treatment described in section 4.3.2. After removal of the liquid phase, 5 mL of 0.02% of NaN_3_ was added to the precipitate and the mixture was boiled for 10 min and cooled. The sample was filtered and injected to the HPSEC (Procedure D). For a parallel set of samples, ileal supernatant after the ethanol treatment was further treated with lichenase or β-xylanase for purification of arabinoxylan and β-glucan (Procedure F) as described in section 4.3.2.

#### 4.3.4. Side Activity of Lichenase and β-Xylanase

Lichenase and β-xylanase were tested for side activity on pure standards of arabinoxylan (wheat arabinoxylan, high viscosity 47 cSt., Megazyme International Ireland) and β-glucan (oat β-glucan, high viscosity 69 cSt., Megazyme International Ireland), respectively under conditions as described in section 4.3.2.

### 4.4. Analysis

#### 4.4.1. Carbohydrates of Fiber Concentrates, Breads and Ileal Supernatants

The starch and total content of NSP of the fiber concentrates, the breads and the corresponding digesta supernatants were measured as described by Bach Knudsen [34] with the modification that the polysaccharides in starch free residue were hydrolyzed with 2 M H_2_SO_4_ for 1 h instead of 1 M H_2_SO_4_ for 2 h. The content of β-glucan in breads was measured by the enzymatic-colorimetric method of McCleary and Glennie-Holmes [35], whereas the β-glucan in β-glucan concentrate and ileal supernatants was determined as non-cellulosic (NCP) glucose by hydrolysis without prior solubilization with 12 M H_2_SO_4_. The arabinoxylan content in fiber concentrates, breads and ileal supernatants was calculated as the sum of arabinose and xylose content obtained by the NSP method. A/X ratio of arabinoxylan was calculated as a quotient of arabinose and xylose content.

#### 4.4.2. Molecular Characterization of Carbohydrates Performed on HPSEC

One hundred and fifty microliters of 1–5 mg/mL concentrated sample was injected for the HPSEC analysis. The high-performance size-exclusion chromatography system Viscotek TDAmax (Malvern Instruments Ltd, Worcestershire, UK) was equipped with a Viscotek automatic injector and pump (VE 2001 GPC Solvent/Sample Module). The sample was eluted at 0.5 mL/min through a series of columns; TSK gel GMPWxl (Tosoh Bioscience Gmbh, Stuttgart, Germany), OH-Pak SB-806 HQ and SB-806M HQ (Shodex, Kanagawa, Japan) using miliQ water with 0.02% NaN_3_ as eluent. The detection system consisted of 90° RALLS and 7° angle LALLS detector with constant optical power output laser diode working at wavelength 670 nm, a four-capillary bridge viscometer (intrinsic viscosity detector) and a refractometer with conventional dual cell (RI), deflection design (Viscotek, Houston, TX, USA). The calibration was done with a pullulan standard of molecular weight 112 kDa (Shodex standard, Showa Denko K.K., Japan). The dn/dc values used were 0.146 mL/g for arabinoxylan and 0.150 mL/g for β-glucan in water solutions [36,37]. The M_w_, proportion of M_w_ <200 kDa and >200 kDa were calculated directly from the chromatograms by use of OmniSEC software (Malvern Instruments Ltd., Worcestershire, UK).

#### 4.4.3. Viscosity of Bread Extracts and Ileal Effluents

The bread after water incubation and *in vitro* and *in vivo* digestion were centrifuged (10,000× *g*, 4 °C, 20 min) in 50 mL tubes and the viscosity of the supernatants was measured in a Brookfield DV-II cone/plate viscometer (Brookfield Engineering Laboratories Inc., Stoughton, MA, USA) at 39 °C at shear rates between 2.25 and 450 s^−1^. The cone of 0.8° angle had a diameter of 4.8 cm. Values of viscosity at shear rate 45 s^−1^ were used.

### 4.5. Statistical Analyses

The M_w_, proportion of M_w_ <200 kDa and >200 kDa of two different extraction methods on five breads were analyzed using a two-way analysis-of-variance model (Table 2). The same response variables obtained from ethanol treated ileal contents were analyzed using a mixed model (Table 3). Moreover, the response variables of the lichenase purified arabinoxylan in the *in vitro* digested WF, GR, RK and AX breads and their ileal supernatant counterparts were analyzed using a one-way analysis-of-variance model (Tables 4 and 5). Log values of viscosity of water extracts and *in vitro* digested breads were calculated by one-way analysis, and of ileal supernatants by a mixed model (Table 6).

All statistical analysis was performed using Statistical Analysis Software (SAS Institute Inc., Cary, NC, USA). All data were expressed as least squares means with differences considered statistically significant at *p* < 0.05.

## 5. Conclusions

*In vitro* and *in vivo* digested breads had a markedly reduced M_w_ compared to water extracted breads and the fiber sources extracted under mild alkali conditions. This was due to the degradation of DF during dough making and baking. Moreover, although the M_w_ was almost similar in the *in vitro* digested GR, RK and AX breads to their ileal supernatant counterparts after the lichenase treatment, their extract viscosity varied substantially between the *in vitro* and *in vivo* digestion depending on the DF content and structure present in bread and ileal extracts. The wheat arabinoxylan present in AX bread appears to be of nutritional interest due to its high concentration in ileal supernatant that contributed to raise intestinal viscosity. This suggests a potential of wheat arabinoxylan in concentrated form for further applications in food manufacturing.

## Figures and Tables

**Table 1:** Content of polysaccharides in the experimental breads (% of DM) and in ileal digesta supernatants (mg/mL).

	Bread					Ileal Supernatant					

	WF	GR	RK	AX	BG	WF	GR	RK	AX	BG	SE
Starch	71.1	58.8	60.8	50.9	60.7	0.4	0.3	0.4	0.4	2.7	0.28
Total NSP	3.5	13.4	13.9	11.2	16.7	19.4	15.9	12.6	56.3	10.6	1.70
soluble	1.7	5.3	5.0	8.4	5.9						
Insoluble	1.7	8.1	8.9	2.8	10.8						
Arabinoxylan	1.7	7.6	7.7	7.5	3.4	15.2	13.9	10.9	44.8	5.3	1.31
Soluble	1.3	3.6	3.7	6.5	1.0						
insoluble	0.3	3.9	4.0	0.9	2.4						
β-glucan *	0.3	2.1	1.9	0.4	4.9	0.2	0.2	0.2	0.9	2.8	0.62
Soluble	0.2	0.6	0.4	0.3	4.0						
insoluble	0.1	1.5	1.5	0.1	0.9						
Cellulose	0.6	1.8	1.8	0.6	6.0	-	-	-	-	-	
A/X of soluble NSP	0.70	0.64	0.70	0.97	1.37	0.77	0.70	0.74	0.97	1.04	0.06

WF, control wheat white bread; GR,dark ground rye bread; RK, kernel rye bread; AX, wheat bread with inclusion of wheat arabinoxylan concentrate; BG, wheat bread with inclusion of oat β-glucan concentrate; NSP, non-starch polysaccharides; A/X, ratio of arabinose to xylose,

* determined as NCP-glucose in ileal supernatant;

SE, standard error.

**Table 2:** Molecular characteristics of water extracted and *in vitro* digested breads.

Treatment	M_w_ (kDa)		<200 kDa		>200 kDa	
WF						
Water extraction	1,627	c	17.1	f	82.9	b
*In vitro* digestion	264	e	82.0	b	18.0	f
GR						
Water extraction	5,479	a	2.1	h	97.9	a
*In vitro* digestion	270	e	74.8	c	25.2	e
RK						
Water extraction	4,411	b	1.9	h	98.1	a
*In vitro* digestion	276	e	75.0	c	25.0	e
AX						
Water extraction	1,015	d	42.1	e	57.9	c
*In vitro* digestion	134	e	93.1	a	6.9	g
BG						
Water extraction	656	d	53.3	d	46.7	d
*In vitro* digestion	240	e	55.7	d	44.3	d
SE	126		1.79		1.79	
Diet	<0.0001		<0.0001		<0.0001	
Method	<0.0001		<0.0001		<0.0001	
Diet*Method	<0.0001		<0.0001		<0.0001	

WF, control wheat white bread, GR, dark ground rye bread; RK, kernel rye bread; AX, wheat bread with inclusion of wheat arabinoxylan concentrate; BG, wheat bread with inclusion of oat β-glucan concentrate; M_w_, weight average molecular weight; <200 kDa, proportion of molecular fraction below 200 kDa (%); >200 kDa, proportion of molecular fraction above 200 kDa (%); Different letters in the same column indicate significant difference at *p* < 0.05.

**Table 3:** Molecular characteristics of ethanol precipitated ileal supernatants of bread-fed pigs.

Bread	M_w_ (kDa)	<200 kDa	>200 kDa
WF	259	82.4	17.6
GR	373	79.9	20.1
RK	366	78.8	21.2
AX	397	87.4	12.6
BG	335	82.9	17.1
SE	69	2.31	2.31
*p value*	0.64	0.13	0.13

WF, control wheat white bread; GR, dark ground rye bread; RK, kernel rye bread; AX, wheat bread with inclusion of wheat arabinoxylan concentrate; BG, wheat bread with inclusion of oat β-glucan concentrate; M_w_, weight average molecular weight; <200 kDa, proportion of molecular fraction below 200kDa (%); >200 kDa, proportion of molecular fraction above 200kDa (%).

**Table 4:** Molecular characteristics of lichenase purified *in vitro* digested breads.

Bread	M_w_ (kDa)		<200 kDa		>200 kDa	
WF	219	c	71.3	a	28.7	c
GR	250	c	62.8	b	37.2	b
RK	514	a	41.6	c	58.4	a
AX	307	b	63.8	b	36.2	b
SE	17		2.11		2.11	
*p value*	<0.0001		<0.0001		<0.0001	

WF, control wheat white bread; GR, dark ground rye bread; RK, kernel rye bread; AX, wheat bread with inclusion of wheat arabinoxylan concentrate; BG, wheat bread with inclusion of oat β-glucan concentrate; M_w_, weight average molecular weight; <200 kDa, proportion of molecular fraction below 200 kDa (%); >200 kD, proportion of molecular fraction above 200kDa (%); Different letters in the same column indicate significant difference at *p* < 0.05.

**Table 5:** Molecular characteristics of lichenase purified ileal supernatants of bread-fed pigs.

Bread	M_w_ (kDa)	<200kDa	>200kDa
WF	360	79.3	20.7
GR	265	74.6	25.4
RK	419	59.8	40.2
AX	315	73.2	26.8
SE	74	4.44	4.44
*p value*	0.49	0.061	0.061

WF, control wheat white bread; GR, dark ground rye bread; RK, kernel rye bread; AX, wheat bread with inclusion of wheat arabinoxylan concentrate; BG, wheat bread with inclusion of oat β-glucan concentrate; M_w_, weight average molecular weight, <200 kDa, proportion of molecular fraction below 200kDa (%); >200 kDa, proportion of molecular fraction above 200 kDa (%).

**Table 6:** Viscosity of extracts after water incubation and *in vitro* and *in vivo* digestion of breads (mean and 95% confidence intervals, mPa.s).

Method of extraction	WF		GR		RK		AX		BG	
Water incubation (Procedure B)	1.3 (1.2–1.4)	e	4.1 (3.8–4.3)	c	4.7 (4.4–4.9)	b	1.8 (1.7–2.0)	d	10.7 (10.1–11.3)	a
*In vitro* digestion (Procedure C)	1.8 (1.7–1.9)	e	5.1 (4.9–5.4)	c	6.6 (6.3–6.9)	b	2.5 (2.4–2.6)	d	10.4 (10.0–10.9)	a
*In vivo* digestion (Procedure D)	5.9 (3.3–10.5)	b	8.4 (4.7–15.0)	b	7.4 (4.2–13.3)	b	15.5 (8.6–27.6)	a	2.6 (1.5–4.7)	c

WF, control wheat white bread; GR, dark ground rye bread; RK, kernel rye bread; AX, wheat bread with inclusion of wheat arabinoxylan concentrate; BG, wheat bread with inclusion of oat β-glucan concentrate; Different letters in the same row indicate significant difference at *p* < 0.05.
